# Supplementary material for: Comparison between School-Age Children with and without Obesity in Nutritional and Inflammation Biomarkers
Source: J Clin Med. 2022 Nov 26;11(23):6973. doi: 10.3390/jcm11236973 (PMC9739253; doi:10.3390/jcm11236973)
Supplement: Supplementary file 1 [file jcm-11-06973-s001.zip › jcm-2032932-supplementary.pdf]

Table S1. Differences in the serum levels of nutritional markers between males and females aged 10–12 years.

	Males n = 87	Females n = 61	p Value [a]
**White blood cells (10e³/µL), mean (SD)**	7.7 (2.4)	7.5 (2.1)	0.7
**Neutrophils (10e³/µL), mean (SD)**	3.6 (1.7)	3.7 (1.5)	0.8
**Lymphocytes (10e³/µL), median (IQR)**	3.0 (1.1)	2.90 (1.1)	0.5 [b]
**Platelets (10e³/µL), median (IQR)**	294.0 (75.0)	292.0 (90.0)	0.6 [b]
**Glucose (mg/dL), mean (SD)**	93.3 (16.2)	93.0 (11.8)	0.9
**Hemoglobin (g/dL), mean (SD)**	12.5 (0.9)	12.4 (0.8)	0.3
**Hematocrit (%), mean (SD)**	37.1 (2.4)	36.8 (2.3)	0.4
**Mean Corpuscular Hemoglobin (pg), median (IQR)**	26.5 (2.3)	27.0 (2.4)	0.2 [b]
**Mean Corpuscular Volume (fL), median (IQR)**	78.5 (5.9)	80.0 (6.6)	0.1 [b]
**Iron (mcg/dL), median (range)**	56.0 (27.0)	68.0 (30.0)	0.04 [b]
**Transferrin (mg/dL), mean (SD)**	280.0 (35.9)	295.4 (36.6)	0.01
**Transferrin-saturated (%), median (IQR)**	15.5 (9.0)	18.2 (9.5)	0.2 [b]
**Ferritin (ng/ml), median (range)**	48.0 (38.5)	37.9 (32.9)	0.01[b]
**Vitamin B12 (pg/ml), mean (SD)**	515.3 (191.8)	545.2 (205.5)	0.4
**Folic Acid (ng/ml), median (IQR)**	6.8 (3.9)	7.7 (4.0)	0.1 [b]
**Uric Acid (mg/ml), median (IQR)**	4.0 (1.8)	4.0 (1.3)	0.7 [b]
**Triglycerides (mg/dL), median (IQR)**	92.0 (78.0)	91.0 (69.0)	0.8 [b]
**Total cholesterol (mg/dL), median (IQR)**	150.0 (31.0)	149.0 (26.5)	0.9 [b]
**HDL cholesterol (mg/dL), median (IQR)**	50.0 (18.0)	47.0 (22.0)	0.4 [b]
**LDL cholesterol (mg/dL), mean (SD)**	73.1 (19.5)	75.9 (20.7)	0.4
**Protein (g/L), mean (SD)**	7.3 (0.3)	7.3 (0.4)	0.8
**Albumin (g/L), median (IQR)**	4.7 (0.3)	4.7 (0.2)	0.8 [b]
**Globulin (g/L), median (IQR)**	2.6 (0.4)	2.6 (0.5)	0.9 [b]
**C-Reactive Protein (mg/L), median (IQR)**	1.1 (4.3)	0.8 (3.5)	0.6 [b]

[a] p value was obtained by the Student's t-test; [b] Mann-Whitney U test. HDL: High-density lipoprotein; IQR: interquartile range; LDL: Low-density lipoprotein; SD: standard deviation.

Table S2. Differences in the serum levels of nutritional markers among children aged 10–12 years, between those with obesity and normal weight.

	**Obesity** *n* **= 61**	**Normal Weight** *n* **= 64**	p **Value** [a]
**White blood cells (10e³/µL), mean (SD)**	8.1 (2.1)	7.2 (2.2)	0.03
**Neutrophils (10e³/µL), mean (SD)**	3.9 (1.4)	3.5 (1.5)	0.1
**Lymphocytes (10e³/µL), median (range)**	3.02 (1.1–5.3)	2.8 (1.0–6.6)	0.005 [b]
**Platelets (10e³/µL), median (range)**	308.0 (13.2–464.0)	282.0 (139.0–512.0)	0.001 [b]
**Glucose (mg/dL), mean (SD)**	93.4 (12.7)	92.1 (16.8)	0.6
**Hemoglobin (g/dL), mean (SD)**	12.3 (0.9)	12.6 (0.8)	0.1
**Hematocrit (%), mean (SD)**	37.1 (2.4)	37.0 (2.3)	0.8
**Mean Corpuscular Hemoglobin (pg), median (range)**	26.2 (18.5–29.7)	27.3 (18.8–30.5)	0.007 [b]
**Mean Corpuscular Volume (fL), median (range)**	79.4 (59.3–86.6)	79.6 (7.2–89.1)	0.5 [b]
**Iron (mcg/dL), median (range)**	52.0 (20.0–116.0)	68.0 (20.0–125.0)	<0.001 [b]
**Transferrin (mg/dL), mean (SD)**	293.3 (39.2)	284.1 (34.3)	0.2
**Transferrin-saturated (%), median (range)**	15.0 (5.0–33.1)	19.4 (5.6–42.2)	<0.001 [b]
**Ferritin (ng/ml), median (range)**	49.8 (13.6–224.2)	40.0 (14.2–99.9)	0.005 [b]
**Vitamin B12 (pg/ml), mean (SD)**	485.0 (163.5)	527.9 (202.5)	0.2
**Folic Acid (ng/ml), median (range)**	6.7 (2.8–17.8)	8.0 (3.4–18.9)	0.1 [b]
**Uric Acid (mg/ml), median (range)**	4.6 (2.0–7.2)	3.5 (1.8–5.9)	<0.001 [b]
**Triglycerides (mg/dL), median (range)**	109.0 (35.0–380.0)	84.0 (35.0–309.0)	0.001 [b]
**Total cholesterol (mg/dL), median (range)**	147.0 (71.0–409.0)	147.5 (96.0–207.0)	0.4 [b]
**HDL cholesterol (mg/dL), median (range)**	42.0 (26.0–66.0)	58.0 (32.0–78.0)	<0.001 [b]
**LDL cholesterol (mg/dL), mean (SD)**	78.1 (22.4)	68.9 (17.9)	0.01
**Protein (g/L), mean (SD)**	7.3 (0.3)	7.2 (0.4)	0.2
**Albumin (g/L), median (range)**	4.6 (4.3–5.0)	4.7 (4.1–5.3)	0.04 [b]
**Globulin (g/L), median (range)**	2.7 (2.0–3.9)	2.5 (2.0–3.6)	0.001 [b]
**C-Reactive Protein (mg/L), median (range)**	3.9 (0.2–30.2)	0.3 (0.0–12.5)	<0.001 [b]

[a] p value was obtained by the Student's t test; [b] Mann-Whitney U test. HDL: High-density lipoprotein; LDL: Low-density lipoprotein; SD: standard deviation.

Table S3. Associations of nutritional and inflammation biomarkers with obesity among children aged 10–12 years.

	**Model 1 Adjusted OR (95% CI)**	***p* Value [a]**	**Model 2 Adjusted OR (95% CI)**	***p* Value [b]**
**Village**				
**Lower SES village**	15.76 (2.75–90.43)	0.002	9.51 (2.04 –44.39)	0.004
**Higher SES village**	Reference		Reference	
**SEX**				
**Males**	2.78 (0.68–11.43)	0.2	2.98 (0.82 –10.88)	0.1
**Females**	Reference		Reference	
**Lymphocytes (10e³/μL)**	2.86 (1.27–6.47)	0.01	2.71 (1.22–5.97)	0.01
**Platelets (10e³/μL)**	1.00 (0.99–1.01)	0.8	1.00 (0.99–1.01)	0.7
**Iron (mcg/dL)**	0.94 (0.91–0.98)	0.001	Not included	
**Transferrin-saturated (%)**	Not included		0.88 (0.79–0.98)	0.02
**Ferritin (ng/ml)**	1.03 (1.00–1.05)	0.05	1.03 (1.005–1.06)	0.02
**Uric Acid (mg/ml)**	1.57 (0.84–2.92)	0.2	2.19 (1.18–4.04)	0.01
**Triglycerides (mg/dL)**	1.01 (1.001–1.03)	0.03	1.02 (1.006–1.03)	0.004
**LDL cholesterol (mg/dL)**	1.05 (1.02–1.09)	0.004	1.03 (0.99–1.06)	0.07
**HDL cholesterol (mg/dL)**	0.88 (0.82–0.94)	<0.001	Not included	
**C-Reactive Protein (mg/L)**	Not included		1.44 (1.11–1.87)	0.006

p value [a] from multivariable logistic regression model 1, Nagelkerke R^2 = 0.74. *p* value [b] from multivariable logistic regression model 2, Nagelkerke R^2 = 0. :0. CI: confidence interval; HDL: High-density lipoprotein; LDL: Low-density lipoprotein; OR: odds ratio; SES: socioeconomic status. Included in the multivariable model were 125 children (61 with obesity).
